# Supplementary material for: Common motor patterns of asymmetrical and symmetrical bipedal gaits
Source: PeerJ. 2021 Aug 16;9:e11970. doi: 10.7717/peerj.11970 (PMC8375508; doi:10.7717/peerj.11970)
Supplement: Supplementary file 1 [file peerj-09-11970-s001.odt]

Comparisions at 1.39 m.s-1, 1.81 m.s-1 and at 2.5 m.s-1

Gait parameters

Figure S1 shows the spatio-temporal parameters of all the analysed gaits. At all speeds stride length (Ls) and stride frequency (Fs) presents differences between all gaits; having skipping the shorter and the most frequent strides as Figure S1(A) and Figure 1S(B) shows.

The duty factor (DF) of the trailing was always less than leading limbs in skipping (Figure 1S(C)). DF during skipping in the leading leg present similar values with running at all speeds. DF in walking showed differences with all gaits and legs (Figure S1(C)).

Figure S1: (A) Stride length, (B) Stride Frequency, (C) Duty factor, (D) and Flight time at different speeds for all gaits. Each point represents individual trials. Lines represent regressions means of bootstrapped data (N = 100), while shaded area indicates a 95% confidence interval of the bootstrapped data.

Muscle synergies

Figure S2 shows the number of synergies required in each experimental condition. When the number of synergies in different conditions were evaluated (Kruskal-Wallis, p<0.001), post-hocs tests did not identify differences (p>0.05 after multiple comparisons). Around 3 synergies, in average, were necessary to meet the reconstruction quality criteria used in this work in all conditions.

Figure S2: Number of synergies (mean ± s.d.) across gaits and speeds.

The modules were composed basically by the same muscles in all gaits. Module 1 included GluM, RF and VL; Module 2 included Triceps Surae muscles; Module 3 covered Tibial muscle; Module 4 comprised BF (Figure S3).

Figure S3: Solid lines in activation timings profiles indicate average and shaded area represent ± s.d. Vertical lines divide the stance (on left) and swing (on right) phases. Motor modules are represented by bars plot. Muscle abbreviations: Gastrocnemius Medius (GM), Soleus (Sol), Rectus Femoris (RF), Vastus Medialis (VM), Tibialis anterior (TA), Biceps Femoris (BF) and Gluteus Medius (GlM).

Figure S4: Cosine similarities (cosim) means (s.d.) across gaits comparisons. * represents mean values of cosim less than similarity threshold (0.6). Abbreviations: Running (R), walking (W), leading leg in skipping (Slead) and trailing leg in skipping (Str).

When muscle weightings were compared across gaits, similarity was observed across all gaits and legs (Figure S4): in most comparisons, 3 of the 4 modules presented highly similar mean values in all conditions (above 0.6); however, synergy 4 appears to be specific to each locomotor pattern. On the other hand, the trailing leg in skipping displayed only one or two shared synergies when compared to walking.

Figure S5: Averaged centers of activation (CoA) ± s.d. of each motor module plotted in polar coordinates. Significant differences between gaits are indicated above each plot. Abbreviations: Running (R), trailing leg in skipping (Str), leading leg in skipping (Slead) and walking (W).

Activation patterns

Figure S5 shows the mean CoA of each module across gaits and speeds. Some significant differences can be observed, namely: walking and running only differ for the temporal pattern of module 2, which appears shifted on the gait cycle; leading skipping and running show similar temporal patterns, with only some differences in the temporal activation of Module 3; when leading leg in skipping is compared to walking, similar temporal patterns in Modules 1 and 2 can be observed; activation of leading skipping appears more similar to that of running than walking; conversely, the temporal pattern of trailing skipping appears very different to those of symmetrical gaits (3 out of 4 CoA were different in the majority of the comparisons).
